# 2296. Trends in reasons for hospital admission among adults ≥18 years hospitalized with laboratory-confirmed SARS-CoV-2 infection—COVID-19-Associated Hospitalization Surveillance Network (COVID-NET), 14 U.S. States, June 2020 – January 2023

**DOI:** 10.1093/ofid/ofad500.1918

**Published:** 2023-11-27

**Authors:** Fiona P Havers, Michael Whitaker, Monica E Patton, Michael Melgar, Art Reingold, Nisha B Alden, Daewi Kim, Kyle P Openo, Andy Weigel, Patricia A Ryan, Libby Reeg, Kathryn Como-Sabetti, Adrienne Domen, Grant Barney, Kevin Popham, Eli Shiltz, Melissa Sutton, H Keipp Talbot, Melanie Crossland, Christopher Taylor

**Affiliations:** CDC, Atlanta, Georgia; CDC, Atlanta, Georgia; CDC, Atlanta, Georgia; Centers for Disease Control and Prevention, Atlanta, Georgia; University of California, Berkeley, Berkeley, CA; Colorado Department of Public Health and Environment, Denver, Colorado; Yale CT Emerging Infections Program, New Haven, Connecticut; Georgia Emerging Infections Program and Atlanta VA Medical Center, Decatur, GA; Iowa Department of Public Health, Des Moines, Iowa; Maryland Department of Health, Baltimore, Maryland; Michigan Department of Health and Human Services, Lansing, Michigan; Minnesota Department of Health, St Paul, Minnesota; University of New Mexico, Emerging Infections Program, Albuquerque, New Mexico; New York State Department of Health, Albany, New York; Universirty of Rochester, Rochester, New York; Ohio Department of Health, Columbus, Ohio; Oregon Health Authority, Portland, Oregon; Vanderbilt University Medical Center, Nashville, Tennessee; Salt Lake County Health Department, Salt Lake City, Utah; CDC, Atlanta, Georgia

## Abstract

**Background:**

During the COVID-19 pandemic, hospitals performed widespread screening for SARS-CoV-2, resulting in identification of asymptomatic or mildly ill persons with SARS-CoV-2 who were admitted for other reasons. We examined trends in reasons for admission to identify hospitalized adults with a positive SARS-CoV-2 test likely admitted for COVID-19-related illness.

**Methods:**

From June 2020–January 2023, hospitalized patients aged ≥ 18 years with a positive screening or clinician-directed SARS-CoV-2 test ≤ 14 days prior to or during hospitalization were identified from > 300 hospitals across 14 states in the population-based COVID-19-Associated Hospitalization Surveillance Network. Trained staff abstracted charts for a representative sample of patients. COVID-19-related admissions were indicated if 1) COVID-19 or 2) COVID-19-associated symptoms or clinical presentations were listed as chief complaint or reason for admission in the history of present illness. Percentages for sampled cases were weighted to account for probability of selection.

**Results:**

Among 424,047 adults hospitalized with SARS-CoV-2 infection, the proportion aged ≥ 75 years increased over time to > 40% by October 2022 (**Figure 1**). The proportion of patients among 34,644 sampled hospitalizations admitted with likely COVID-19-related illness declined from 88.0% (June 1, 2020–June 19, 2021) to 72.4% (June 19, 2022–January 31, 2023) (**Figure 2**). The greatest decline in COVID-19-related admissions occurred in adults aged 18–49 years; the smallest decline occurred in adults aged ≥75 years: > 80% of these admissions in all periods were likely COVID-19-related. Among adults hospitalized from June 2022–January 2023, 27.1% had non-COVID-19 reasons for admission, including planned procedures (2.4%), obstetrics labor and delivery (5.0%), psychiatric admission requiring acute medical care (2.5%), trauma (2.6%), and other reasons likely unrelated to COVID-19 (14.6%).

**Figure 1.**
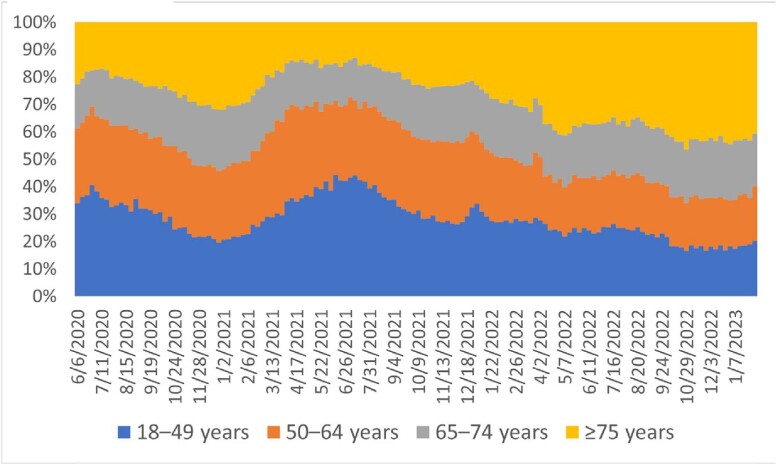
Proportion of laboratory-confirmed SARS-CoV-2 infection-associated hospitalizations by adult age group, June 2020 – January 2023

**Figure 2.**
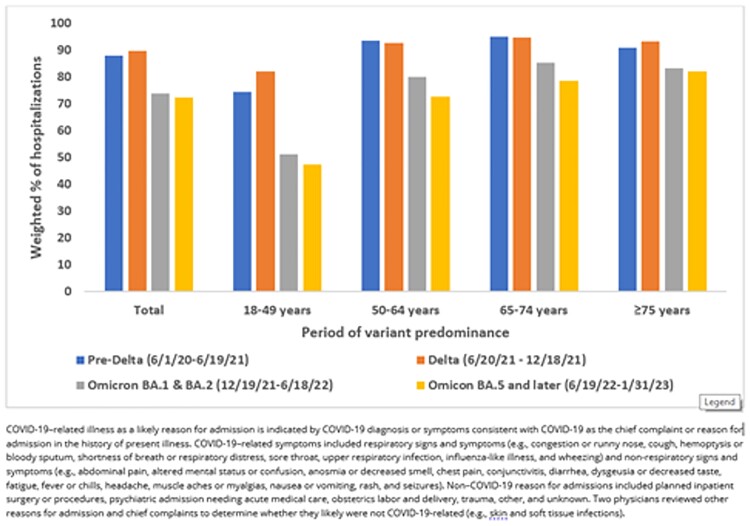
Proportions of hospitalized adults with laboratory-confirmed SARS-CoV-2 infection who had COVID-19 as a likely reason for admission, by age group and variant predominance period — COVID-NET, June 2020 – January 2023

**Conclusion:**

While the proportion of COVID-19-related admissions decreased over time among patients hospitalized with laboratory-confirmed SARS-CoV-2, most adults with a positive SARS-CoV-2 test were likely admitted for reasons related to COVID-19. Misclassification of admission reason may occur.

**Disclosures:**

**Art Reingold, MD**, GSK: Advisor/Consultant|Takeda: Advisor/Consultant|VBI: Advisor/Consultant

